# The Anticoagulation of Calf Thrombosis (ACT) project: study protocol for a randomized controlled trial

**DOI:** 10.1186/1745-6215-13-31

**Published:** 2012-04-02

**Authors:** Daniel Horner, Kerstin Hogg, Richard Body, Michael J Nash, Kevin Mackway-Jones

**Affiliations:** 1 Emergency Department, Manchester Royal Infirmary, Central Manchester NHS Foundation Trust, Oxford Road, Manchester M13 9WL, UK; 2 The University of Manchester, Oxford Road, Manchester M13 9PL, UK; 3 Thrombosis Group, The Ottawa Hospital, 1053 Carling Avenue, Ottawa Ontario K1Y4E9, Canada; 4 Haematology Department, Central Manchester NHS Foundation Trust, Oxford Road, Manchester M13 9WL, UK

**Keywords:** Anticoagulants, embolism, lower extremity, venous thrombosis

## Abstract

**Background:**

Half of all lower limb deep vein thrombi (DVT) in symptomatic ambulatory patients are located in the distal (calf) veins. While proximal disease warrants therapeutic anticoagulation to reduce the associated risks, distal DVT often goes untreated. However, a proportion of untreated distal disease will undoubtedly propagate or embolize. Concern also exists that untreated disease could lead to long-term post thrombotic changes. Currently, it is not possible to predict which distal thrombi will develop such complications. Whether these potential risks outweigh those associated with unrestricted anticoagulation remains unclear. The Anticoagulation of Calf Thrombosis (ACT) trial aims to compare therapeutic anticoagulation against conservative management for patients with acute symptomatic distal deep vein thrombosis.

**Methods:**

ACT is a pragmatic, open-label, randomized controlled trial. Adult patients diagnosed with acute distal DVT will be allocated to either therapeutic anticoagulation or conservative management. All patients will undergo 3 months of clinical and assessor blinded sonographic follow-up, followed by 2-year final review. The project will commence initially as an external pilot study, recruiting over a 16-month period at a single center to assess feasibility measures and clinical event rates. Primary outcome measures will assess feasibility endpoints. Secondary clinical outcomes will be collected to gather accurate data for the design of a definitive clinical trial and will include: (1) a composite endpoint combining thrombus propagation to the popliteal vein or above, development of symptomatic pulmonary embolism or sudden death attributable to venous thromboembolic disease; (2) the incidence of major and minor bleeding episodes; (3) the incidence of post-thrombotic leg syndrome at 2 years using a validated screening tool; and (4) the incidence of venous thromboembolism (VTE) recurrence at 2 years.

**Discussion:**

The ACT trial will explore the feasibility of comparing therapeutic anticoagulation to conservative management in acute distal DVT, within a modern cohort. We also aim to provide contemporary data on clot propagation, bleeding rates and long-term outcomes within both groups. These results will inform the conduct of a definitive study if feasibility is established.

**Trial registration:**

Current Controlled Trials ISRCTN75175695

## Background

Venous thromboembolic (VTE) disease is an international, topical and costly healthcare burden. Incidence rates are equivalent to that of stroke within the western hemisphere [1] and disease consequences can be as severe. Recent studies addressing prognosis provide a stark reminder of continuing poor outcome, quoting a 15% mortality rate at 3 months post diagnosis for VTE involving the pulmonary vascular tract [2]. Outcome from VTE confined to the lower extremities fares little better, with a reported short-term all-cause mortality between 7% and 15% [3]. Observational data suggests reduced survival compared to control subjects after first episode of symptomatic deep vein thrombosis. This trend has been shown to persist for up to 8 years post diagnosis [4]. Clinical research demonstrating poor outcome has led to a national focus on early diagnosis and active prevention, with the creation of guidelines from both the UK Health Technology Assessment (HTA) group and the National Institute of Clinical Excellence (NICE) within the last decade [5,6].

Despite the large body of research on VTE, controversy still remains regarding many aspects of therapeutic clinical practice. One such area is that of distal deep vein thrombosis (DVT), a condition previously thought to be of limited clinical significance. There are multiple epidemiological studies suggesting distal thrombi constitute approximately 50% of objectively diagnosed lower limb disease in symptomatic ambulatory patients [7-9]. This proportion may be even higher in asymptomatic disease or hospitalized patients [10]. However, the benefits of intervention in distal disease remain poorly researched, with conflicting international guidance on investigation and treatment.

Some authors question the ability of ultrasound to diagnose distal DVT. Indeed, recent meta-analyses have consistently failed to show a pooled sensitivity for detection of distal thrombosis by ultrasound any higher than 75% [11,12]. This failing could well be related to the potential pitfalls of the current gold standard: contrast venography has been noted as a potential cause of DVT and has many additional caveats, including extravasation reactions, technical limitations and variable interobserver reliability [13,14]. Despite these failings, many clinicians cite the poor sensitivity data and choose to base their management strategies on serial compression ultrasound of the thigh, avoiding the distal veins altogether. In the absence of sonographic progression to proximal veins after 7 days, the presence of distal disease is presumed to be clinically irrelevant. Recent well conducted studies report a non-significant difference in 3-month VTE event rates between patients randomized to be investigated by serial or complete leg ultrasound in suspected DVT [15,16]. The British Society of Haematology endorse this approach to suspected lower limb VTE in a national guidance document [17]. Thus, many clinicians withhold anticoagulation after serially negative proximal ultrasound.

Conversely, it is also well recognized that a proportion of untreated distal disease will propagate, embolize and/or lead to chronic venous pathology. Current estimates of proximal propagation in untreated patients range between 0% and 29%, with some untreated patients developing pulmonary emboli during short-term follow-up [18]. The most relevant studies assessing complication rates in untreated patients can be seen in Table 1[19-25]. There have also been previous reports of fatal pulmonary embolism occurring within the 7 days after initial negative proximal ultrasound in suspected disease [26]. The potential to cause post-thrombotic syndrome (PTS) is valid but as yet unquantified [27,28]. These sequelae prompt some clinicians to advocate standard therapeutic anticoagulation for all. Several international organizations endorse this approach when diagnosis is clarified [29-31].

**Table 1:** Prospective studies assessing complication rates in untreated distal deep vein thrombosis (DVT) patients

Author/year	Population	Sample size	Diagnostic method	Duration of follow-up for primary endpoint	VTE complication rate
Schwarz et al. 2010 [22]	Low-risk ambulatory patients with isolated calf muscle thrombus	53	CUS	3 months	2/53 = 3.77%

Palareti et al. 2010 [21]	Symptomatic outpatients	65	CUS	3 months	5/64 = 7.8%

Macdonald et al. 2003 [19]	Mostly symptomatic surgical and medical inpatients (68.6%) with isolated calf muscle vein thrombus	135	CUS	3 months	4/135 = 3%

Schwarz et al. 2001 [23]	Symptomatic outpatients with isolated calf muscle vein thrombosis	32	CUS	3 months	8/32 = 25%

Lohr et al. 1995 [18]	Mostly symptomatic surgical and medical inpatients (59.4%)	192	CUS	4 weeks	21/169 = 12.4%

Oishi et al. 1994 [20]	Asymptomatic postoperative total hip replacement/total knee replacement patients	41	CUS	12 months	7/41 = 17.1%

Lagerstedt et al. 1985 [17]	Symptomatic medical patients	28	Isotopic uptake confirmed by ascending phlebography	90 days	8/28 = 29%

CUS = compression ultrasound; VTE = venous thromboembolic complication rate: this refers to ascending proximal extension of the thrombus to the popliteal vein or development of symptomatic pulmonary embolism, except for the study by Schwarz et al. [23]. In this trial, patients were commenced on therapeutic anticoagulation if the distal thrombus propagated to any of the deep calf veins. Many cases were therefore treated prior to potential popliteal extension.

It remains unclear whether the benefits of treatment outweigh the potential harms. The only randomized trial comparing conservative management to standardized oral anticoagulation in distal DVT was performed by Lagerstedt et al. in 1985 [19]. A total of 51 participants were included. The authors demonstrated a 29% 3-month recurrence rate and a 32% 1-year recurrence rate for conservatively managed patients with distal DVT. The incidence of recurrence in warfarinized patients was significantly lower, 0% at 3 months and 4% at 1 year. The results from this trial have been much debated, with many authors highlighting the small sample size, composite diagnostic standards and unequal baseline characteristics between groups [32].

Recent studies using ultrasonography to detect recurrence or propagation have failed to replicate Lagerstedt et al.'*s *data. Using a limited treatment regimen of reduced dose heparin for 4 weeks only, Parisi et al. demonstrated a 2.9% propagation rate at 3-month follow-up [33]. The blind, prospective CALTHRO study has recently reported low rates of venous thromboembolism/recurrence in untreated patients at 3 months, noting an event rate of 7.8% (95% CI 3% to 17%) [23]. Schwarz et al. have demonstrated further reduced event rates when selecting out low-risk distal DVT for conservative treatment, with propagation in only 3.7% of untreated patients [24]. However, no study has attempted to definitively answer the question by performing an adequately powered prospective randomized controlled trial (RCT). This is highlighted by a recent meta-analysis that notes the heterogeneity of trial data and fails to provide a robust conclusion, despite analyzing data from over 450 patients [34]. Equipoise remains, perhaps best highlighted by recent European research noting the profound and continuing regional variability in diagnostic and therapeutic approach to distal DVT [35]. Recent articles have highlighted the need for robust evidence and called urgently for further prospective RCT data to inform clinical decision making [32,36,37].

We designed a trial to examine the feasibility of testing the applicable null hypothesis: that therapeutic anticoagulation for 3 months confers no significant clinical benefit in the management of acute symptomatic distal DVT, when compared to conservative treatment alone.

## Methods

### Study aims

The Anticoagulation of Calf Thrombosis (ACT) trial aims to compare the incidence of venous thromboembolic complications in patients with distal deep vein thrombosis treated with either standard therapeutic anticoagulation or conservative management.

### Study design and setting

**Figure 1:**
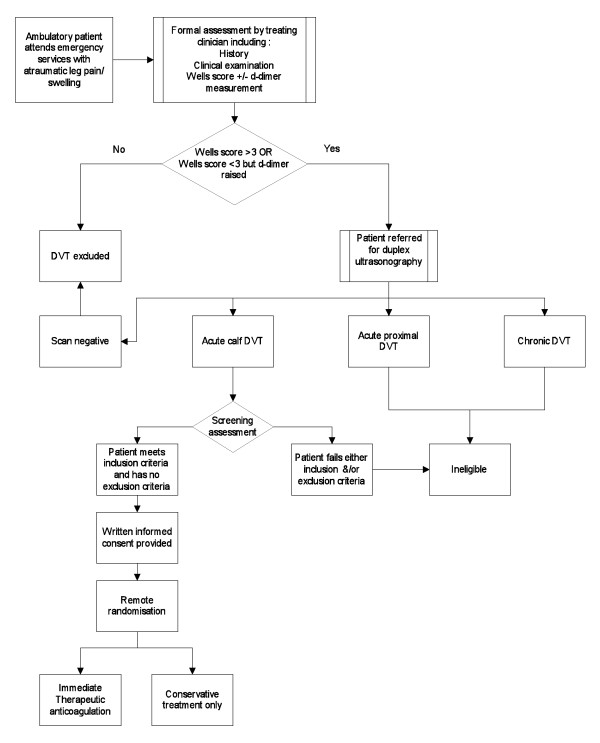
**Summary of trial design/patient flow**. Proximal DVT relates to acute thrombotic disease above the level of the trifurcation of the popliteal vein. Chronic DVT relates to any reported thrombosis detected on prior documented ultrasound, previously treated, or with chronic appearance on contemporary ultrasound exam. DVT = deep vein thrombosis.

The study will be initially conducted as a prospective, randomized, open-label, pragmatic, controlled trial within the Emergency Department (ED) at Central Manchester University Hospitals NHS Foundation Trust. The ED has an average annual attendance figure of 110,000. The trial will begin as an external pilot project, recruiting distal DVT patients over a 16-month period at a single center to assess feasibility and gather accurate clinical outcome data. A study process flow chart is given in Figure 1.

### Ethical considerations

Ethical approval for this study has been obtained from the North West Greater Manchester Central Research Ethics Committee (ref: 10/H1008/97) as a Controlled Trial of an Investigative Medicinal Product (CTIMP). The Medicines and Healthcare products Regulatory Agency (MHRA) have granted clinical trial authorization. A Data Safety and Monitoring Board (DSMB) including a leading expert in thrombosis/hemostasis will be convened to evaluate data and comment on safety within the trial. A Trust Steering committee will oversee local trial conduct and governance. The study is subject to all ongoing NHS Research and Development governance checks regarding CTIMP projects.

### Study interventions

Patients randomized to trial group A will receive immediate therapeutic anticoagulation with initial daily administration of subcutaneous low molecular weight heparin (LMWH), followed by 3 months oral standard NHS anticoagulant pharmacotherapy. Pending UK adoption of novel agents, oral anticoagulation will be achieved with warfarin targeted to an international normalized ratio (INR) of 2.5 (range 2.0 to 3.0). Patients will be monitored by a dedicated anticoagulant clinic at the study site and seen at regular intervals for INR testing. Methods for adjusting warfarin dose and maintaining target INR will be at the discretion of the anticoagulant clinic service. Compliance will be evaluated within treatment groups. Group A patients will also receive grade 2 compression hosiery fitted externally through the orthotics department.

Group B trial patients will receive symptomatic treatment only, in the form of simple analgesia and fitted grade 2 compression hosiery.

### Identification of eligible patients

All ambulatory patients with suspected DVT attending the ED will undergo risk stratification, blood investigation and subsequent complete lower limb duplex compression ultrasound (CUS), in line with standard practice. Prior to ultrasonography, all patients with suspected DVT will be provided with a patient information sheet outlining the trial protocol.

Patients will be managed in an ED thrombosis clinic the same day, where they will be further counseled regarding diagnosis and treatment options. The presence of isolated thrombus in any of the peroneal, soleal, gastrocemial, or tibial veins on duplex CUS detected by an accredited vascular technician, will constitute the diagnosis of distal DVT. Patients with confirmed distal disease will be screened for eligibility by a trained researcher. Inclusion/exclusion criteria are documented below. Only patients able to provide written informed consent will be approached for inclusion. Demographic data will be collected on case report forms, including risk factors (permanent and temporary), provocation, baseline blood tests and examination findings.

#### Inclusion criteria

• Aged 16 or above

• Symptomatic attendance to the Emergency Department with atraumatic leg pain and/or swelling as the principal complaint

• Objective diagnosis of distal deep vein thrombosis by duplex vascular ultrasound

#### Exclusion criteria

• Hospitalised patients (all inpatients)

• Long term therapeutic anticoagulation

• Associated confirmed venous thromboembolic disease (Proximal leg DVT, PE or central vein thrombosis)

• Contraindication to anticoagulation (presence of active bleeding, recent haemorrhagic stroke or upper gastrointestinal bleed)

• Active cancer

• Any other indication for anticoagulation according to national/local guidance: prior confirmed and treated above knee DVT/PE, antiphospholipid syndrome or symptomatic inherited thrombophilia.

• Pregnancy

• Chronic non propagating thrombus

• Previous enrollment to the ACT trial

### Randomization technique

Randomization will occur after patient consent has been taken. Participating patients will be assigned to one of two groups by a remote, computerized, web-based randomization sequence, constructed with variable permuted block size. Group A will be allocated to receive therapeutic anticoagulation with standard pharmacotherapy, group B to receive conservative management. All patients will be briefed in person and writing regarding the clinical signs of extending DVT/PE and advised to contact the trial team or return to the ED with any concerns.

### Blinding

This is an open-label study. Although previous trials have used 'sham' anticoagulant clinics we feel use of placebo and frequent hospital visits to maintain blinding would be potentially unethical and deleterious to recruitment. Complications in the context of warfarinization also need urgent treatment and an unblinding protocol would naturally delay this.

All ultrasonographers will be blinded to allocation for repeat scans. Clinical outcome measures are primarily objective, which should minimize the risk of measurement bias.

### Patient follow-up procedures

Patients will return at 7 and 21 days for follow-up duplex CUS and clinical review. Vascular radiology technicians will be blinded to treatment allocation for all scans. Propagation of DVT to the level of the popliteal vein (above the trifurcation) at any point post randomization will be considered as proximal extension and result in immediate therapeutic anticoagulation. Patients will be clinically reviewed and outcome data collected when they attend for repeat CUS. Worsening symptoms in the context of non-propagation above the trifurcation will be assessed carefully and further investigations will be dictated by clinical need.

At the end of the 3-month treatment period all subjects will be followed up via medical record review and structured telephone interview. An ED appointment will be arranged if any queries or clinical concerns persist. All patients will be encouraged to continue wearing compression stockings daily for 2 years, as per current evidence [38]. Suspicion of pulmonary VTE at any stage will be investigated as per current practice and confirmed by Prospective Investigation Of Pulmonary Embolism Diagnosis (PIOPED) reported V/Q scan or computed tomography (CT)-pulmonary angiography [39]. Any patient diagnosed with pulmonary VTE will receive immediate therapeutic anticoagulation as per current practice. Out of normal working hours, patients will be advised to attend the ED with any concerns, where a protocol for investigation of suspected pulmonary embolism in ambulatory patients is already standard practice.

Final clinical review and data collection will occur at 2 years post inclusion, regarding the incidence and severity of post thrombotic syndrome and the incidence of DVT recurrence in all patients. The diagnosis and severity of PTS will be assessed using the standardized scoring system validated by Villalta et al. [40].

### Patient outcome measures

As an external pilot study, the primary endpoints for the trial will constitute measures of feasibility only. A successful pilot RCT seeks to collect data regarding process, resources, management and scientific data [41]. Feasibility outcomes have been designed to reflect this. Clinical measures of treatment effect and safety will be recorded as secondary outcomes, in order to inform further sample size calculations and data inference for potential future multicenter research.

Primary feasibility outcomes are: incidence of the index condition, the proportion of eligible patients within the screening cohort, recruitment rate for those deemed eligible, allocation crossover and short-term compliance with the study protocol.

Secondary outcomes are: combined incidence of thrombus propagation to the popliteal vein, DVT recurrence, development of pulmonary embolism or VTE related sudden death during the 3-month intervention period; incidence of major and minor bleeding episodes during the 3-month treatment period; incidence of post-thrombotic leg syndrome at 2 years; and incidence of VTE recurrence at 2 years.

Outcome measures will be defined using the following tools: DVT recurrence or development of pulmonary embolism will be confirmed by objective diagnostic criteria, either via repeat CUS in the presence of worsening symptoms or PIOPED reported ventilation-perfusion scan or CT pulmonary angiogram in the presence of new chest symptoms [39]. Any cases of sudden death during the interventional phase of the trial will be assessed by a panel of experts blinded to treatment allocation, including a Professor of Emergency Medicine, Consultant Hematologist and Consultant Respiratory Physician. A consensus decision will be required regarding VTE as the principal cause of death.

Major bleeding episodes will be defined as standardized in 2005 by Schulman et al.: clinically overt and associated with a fall in hemoglobin of 20 g/l, resulting in the need for transfusion of two or more units of red cells, involving a critical site, or fatal [42]. Minor bleeding episodes will be subcategorized as per Schulman et al. in 2009 into clinically relevant, or nuisance bleeding [43].

Post-thrombotic syndrome will be diagnosed and numerically graded using the validated and internationally adopted Villalta scale [40].

### Withdrawal, allocation crossover and protocol violation

Participants withdrawing from the study voluntarily will be included in the intention to treat analysis. Allocation crossover will be deemed to occur if patients allocated to conservative treatment are prescribed full dose therapeutic anticoagulation for > 5 days at any stage during 3-month follow-up, or patients allocated to anticoagulation have therapy withheld for > 5 days.

### Data safety and monitoring board (DSMB)

The DSMB will be independent and composed of three principal members: a leading expert in thrombosis/hemostasis, an independent statistician and an expert in clinical trials (chair). All pharmacovigilance reports including serious adverse events, adverse events, protocol violations and allocation crossovers will be reported to the DSMB along with all clinical endpoint data collected. The group will be convened after recruitment of 50 patients. No criteria exist for early termination of the pilot study; judgment of the DSMB will be acknowledged and followed.

The board comprises: Professor Henry Kitchener (Chair), honorary consultant gynecological oncologist and chair of the National Cancer Research Institute's Gynaecological Clinical Studies Group; Dr Trevor Baglin, consultant hematologist and President of the British Society for Haemostasis and Thrombosis; and Dr Steve Roberts, medical statistician and senior lecturer at the University of Manchester.

### Sample size considerations

The most recent prospective evidence estimates the 3-month composite risk of VTE in untreated patients with undifferentiated distal DVT to be approximately 5% [23]. Data from separate research cites the above risk to be 1% in patients receiving anticoagulation [16]. To achieve this expected difference between groups, 489 patients per group would provide statistical power of 80% with a two-sided α of 0.05. For a definitive study, the required sample size is thus currently estimated at approximately 1,000 patients.

For the primary feasibility study we will recruit over a 16-month period initially, aiming to achieve roughly 10% of the current sample size estimate at 100 patients. An updated power calculation will be derived from the primary feasibility data along with refinements to trial design for use in the definitive RCT.

### Statistical analysis

As a feasibility study, principal analysis will focus on the incidence of distal DVT as the index condition within the screening cohort and the proportion of eligible patients willing to participate in the trial. Protocol violations and allocation crossover rate will also be assessed within the two groups to determine the feasibility of maintaining treatment allocation within each cohort for the duration of the study period. Binomial confidence intervals will be estimated for all proportions using the Wilson score exact method.

The predefined criteria for assessing success of feasibility will constitute the following: (1) index disease incidence > 5% within the screening cohort, (2) > 70% recruitment rate within eligible participants and (3) < 25% protocol violation rate.

The secondary analysis will be a comparison of anticoagulation versus conservative treatment for prevention of the secondary clinical endpoint following the 'intention to treat' principle. A further 'per protocol' analysis of all clinical endpoints will take place excluding all withdrawals, allocation crossovers and protocol violations. Proportions will be compared for statistical significance using Fisher's exact test and a further descriptive analysis made of the individual components forming the composite primary outcome. An estimate will be made with 95% confidence interval of absolute risk reduction. Together with the primary feasibility outcomes, this data will allow estimation of the number of sites, duration of recruitment and resources needed to conduct the definitive multicenter study.

Further intention to treat analyses of secondary and tertiary endpoints occurring within the two groups will be compared using Fisher's exact test. All significance tests will be two sided.

## Discussion

Management of isolated distal DVT is controversial throughout the developed world. Investigation and treatment strategies continue to vary locally and internationally. National management guidance continues to change based on emerging evidence [44,45]. These guidelines acknowledge the deficit in the literature and modern papers continue to call for prospective clinical trials [18,32,36,46]. There is a pressing and documented need to clarify the benefits of any treatment and the risks involved.

The ACT study has begun as a feasibility project, recruiting over a 16-month period. The main clinical outcomes assessed will incorporate both VTE-related and anticoagulant-related complications. Analysis of feasibility data will support future sample size calculations, allow refinement of methodology and inform the conduct and coordination of an adequately powered multicenter RCT.

Selection of the most appropriate primary outcome for the definitive trial is scientifically challenging. We propose a composite primary outcome of VTE-related death, DVT propagation, pulmonary embolism or major bleeding occurring within 3 months. This outcome combines the most relevant considerations for clinicians facing the decision of whether to prescribe anticoagulation for a patient with isolated distal DVT. Acknowledging the current equipoise, this composite outcome focuses on net benefit, balancing the risks of withholding anticoagulation against the risks of prescribing anticoagulation.

Each component of this composite outcome is directly relevant to our research question. While death is arguably the most important outcome, it is not the only consideration in the decision to anticoagulate. Proximal propagation is a proxy marker for aggressive disease and, due to the potential for death and pulmonary embolism, it would be unethical to continue to withhold anticoagulation in its presence. Major hemorrhage is the main concern with therapeutic anticoagulation and largely responsible for our situation of equipoise.

The use of composite outcomes within controlled trials is supported by international bodies [47]. Advantages include the engagement of multiplicity and derivation of a clinically important result from a smaller sample, with consequent reduction in costs and timely introduction of appropriate treatments. Modern interventional trials in venous thromboembolic disease continue to rely on a composite of endpoints as the primary outcome [43,48]. Disadvantages include dilution of treatment effect, the detrimental impact of subjective outcomes and the equal weighting that is given to factors of varying importance to patients and clinicians [49,50]. We aim to address these concerns as follows: (1) our composite outcome includes primarily objective measures, (2) the composite outcome includes only those factors that would directly influence the decision to anticoagulate and are therefore crucial to definitively answer our research question, and (3) all individual features of the composite endpoint will be separately identified within the secondary clinical outcomes to allow direct and transparent statistical comparison between groups.

In tandem with the use of a composite outcome we will also involve a health economist within the definitive trial design/analysis, to assess economic merits and overall health utility of the research question.

Other bleeding events, VTE recurrence and the development of post-thrombotic syndrome will constitute additional secondary outcomes. With a large dataset, analysis can also extend to search for individual factors significantly associated with propagation within the conservatively treated cohort. This can be achieved using regression techniques to examine elements within the history, examination and workup that can subsequently be classed as predictive of adverse outcome. If significant predictors exist, consideration can be given to development of a decision tool aimed at helping clinicians to decide which distal thrombi to anticoagulate. This research is essential in developing an ideal model of risk stratification and individualized treatment [36]. Therapeutic anticoagulation should be tailored to those at risk.

A definitive answer to the management questions surrounding investigation and treatment of distal DVT has huge implications for both patients and clinicians. If a real and significant reduction in risk of complications is seen with therapeutic anticoagulation, diagnostic strategy and clinical guidance can become focused and coherent both nationally and internationally. All patients can subsequently receive evidence-based therapy aiming to prevent both short and long-term complications of disease. If the absolute risk reduction seen is deemed non-significant, or benefit limited by bleeding risks, then clinicians can pursue conservative management with confidence. Anticoagulation can be restricted in the majority of cases, resulting in reduced healthcare costs and bleeding complications. Either way, the ACT study aims to benefit both patients and clinicians by providing modern evidence to assist decision making for this challenging and relatively common clinical scenario.

### Trial status

The ACT trial was conceived and designed in 2009, with successful application for peer reviewed funding through the College of Emergency Medicine (UK) in 2010 and National Institute for Health Research (NIHR) portfolio adoption in 2011. Recruitment to the trial began in January 2011. As of 15 January 2012, 62 patients have been successfully recruited within a 12-month period. Steering committee review has occurred with governance oversight and full approval for continued recruitment to the end of the feasibility window. Recruitment is planned to continue until the end of April 2012. Following protocol completion and subsequent analysis, preliminary results will be available towards the end of the year.

## Competing interests

The authors declare that they have no competing interests.

## Authors' contributions

KH, KM-J and DH were responsible for identifying the research question and contributing to drafting of the initial protocol. RB and MJN both contributed to the development of the protocol and study design, as members of the research group. DH was responsible for the drafting of this paper, although all authors provided comments on the drafts and read and approved the final version.
